# Chidamide Inhibits Aerobic Metabolism to Induce Pancreatic Cancer Cell Growth Arrest by Promoting Mcl-1 Degradation

**DOI:** 10.1371/journal.pone.0166896

**Published:** 2016-11-22

**Authors:** Mu He, Zhixin Qiao, Yanbing Wang, Qiyuan Kuai, Changlan Li, Yu Wang, Xingwei Jiang, Xuanlin Wang, Weijing Li, Min He, Suping Ren, Qun Yu

**Affiliations:** 1 Department of Blood Products and Substitutes, Beijing Institute of Transfusion Medicine, Beijing, China; 2 Microbiology Laboratory, Shunyi District Center for Disease Control and Prevention, Beijing, China; 3 Medical Research Centre, Beijing Tongren Hospital, Capital Medical University, Beijing, China; University of South Alabama, UNITED STATES

## Abstract

Pancreatic cancer is a fatal malignancy worldwide and urgently requires valid therapies. Previous research showed that the HDAC inhibitor chidamide is a promising anti-cancer agent in pancreatic cancer cell lines. In this study, we elucidate a probable underlying anti-cancer mechanism of chidamide involving the degradation of Mcl-1. Mcl-1 is frequently upregulated in human cancers, which has been demonstrated to participate in oxidative phosphorylation, in addition to its anti-apoptotic actions as a Bcl-2 family member. The pancreatic cancer cell lines BxPC-3 and PANC-1 were treated with chidamide, resulting in Mcl-1 degradation accompanied by induction of Mcl-1 ubiquitination. Treatment with MG132, a proteasome inhibitor reduced Mcl-1 degradation stimulated by chidamide. Chidamide decreased O_2_ consumption and ATP production to inhibit aerobic metabolism in both pancreatic cancer cell lines and primary cells, similar to knockdown of Mcl-1, while overexpression of Mcl-1 in pancreatic cancer cells could restore the aerobic metabolism inhibited by chidamide. Furthermore, chidamide treatment or Mcl-1 knockdown significantly induced cell growth arrest in pancreatic cancer cell lines and primary cells, and Mcl-1 overexpression could reduce this cell growth inhibition. In conclusion, our results suggest that chidamide promotes Mcl-1 degradation through the ubiquitin-proteasome pathway, suppressing the maintenance of mitochondrial aerobic respiration by Mcl-1, and resulting in inhibition of pancreatic cancer cell proliferation. Our work supports the claim that chidamide has therapeutic potential for pancreatic cancer treatment.

## Introduction

Pancreatic cancer, one of the most lethal cancers has an extremely poor prognosis due to its aggressive nature, the 5-year relative survival rate is less than 6%[1]. In the US, the American Cancer Society estimates that there will be 53,070 new pancreatic cancer cases and 41,780 deaths in 2016[2]. Unfortunately, only 20% of patients with pancreatic cancer are candidates for surgery, with approximately 80% showing metastasis at the initial diagnosis[3], and present chemotherapy and radiotherapy are only marginally efficacious in pancreatic cancer. Gemcitabine based therapy which is the first line treatment for advanced pancreatic cancer, has a poor response rate[4]. Pancreatic cancer therefore remains a challenge to treat with well-tolerated and effective chemotherapies.

A recent study showed that the function of mitochondria in tumor cells was intact and that these mitochondria have no defects compared with normal cells. Mitochondrial oxygen metabolism is still an important source of ATP in tumor cells to maintain an energy supply for rapid growth[5, 6]. In pancreatic cancer cells, more than 75% of the ATP is product by oxidative phosphorylation analyzed by seahorse assay (data not shown). Mcl-1, as an anti-apoptotic member of the Bcl-2 family, is highly expressed in human pancreatic cancer[7]. And another function for Mcl-1 in regulating mitochondrial respiration and dynamics was revealed[8]. The matrix-localized Mcl-1 maintains mitochondrial cristae morphology and supports oxidative phosphorylation and ATP production[8]. Strategies to inhibit both mitochondrial and anti-apoptotic functions of Mcl-1 may synergize by restricting cancer cell proliferation and activating cell death[9].

Histone deacetylases (HDACs) modify the structure of histone and non-histone proteins through altering the acetylation status[10]. Abnormally high class I HDAC expression has been found in pancreatic cancer[11]. HDAC inhibitors have been developed for therapeutic intervention in recent years[12]. Chidamide (Epidaza) is a novel benzamide HDAC inhibitor that was approved by China Food and Drug Administration (CFDA) for the treatment of peripheral T-cell lymphoma (PTCL) in December 2014[13]. At present, chidamide is undergoing clinical trials in the United States and China for treatment of solid tumors[14]. Recent studies have found an anti-cancer effect of chidamide in pancreatic cancer cells[15]. Our previous study demonstrates that chidamide, in combination with gemcitabine. causes growth inhibition of pancreatic cancer cells by inducing p21 expression and cell cycle arrest, and down-regulates the expression of Mcl-1[16]. In this study, we clarify the mechanism of Mcl-1 degradation, investigate the metabolic role of Mcl-1 and propose one of the underlying molecular mechanisms of chidamide in pancreatic cancer.

## Materials and Methods

### Chemicals, reagents and antibodies

Chidamide was synthesized by Shenzhen Chipscreen Biosciences Ltd. (Shenzhen, China) and dissolved in dimethyl sulfoxide (DMSO). MG132 was purchased from Sigma-Aldrich (St. Louis, MO, USA). Primary antibody anti-Mcl-1 was purchased from Abcam^®^ (Cambridge, MA, USA), anti-Acetylated-Lysine and anti-β-actin were obtained from Cell Signaling Technology (Boston, MA, USA), and anti-ubiquitin was purchased from Santa Cruz Biotechnology Inc (Santa Cruz, CA, USA).

### Cell culture

Human pancreatic cancer cell lines (BxPC-3 and PANC-1) were provided by Shanghai Institute of Biochemistry and Cell Biology, Chinese Academy of Sciences (Shanghai, China). Primary human pancreatic cancer cells (pdc0001 and pdc0015) were provided by the National Center for Nanoscale Science (Beijing, China). The cell lines were maintained at 37°C in 5% CO_2_ in DMEM supplemented with 10% fetal bovine serum, penicillin/streptomycin (100 U/ml each) and NaHCO_3_ (2 g/L).

### Western blot

Cells were washed and lysed in RIPA lysis buffer, protein lysates concentrations were measured by BCA protein assay, and equal amounts of proteins were separated by 12% SDS-PAGE gel and transferred onto PVDF membranes (Millipore, Billerica, MA, USA). After blocking with 5% skim milk for 1 h, the PVDF membranes were probed with corresponding antibodies.

### Quantitative real-time PCR

Total RNA was extracted with Trizol (Invitrogen, MA, USA) and reverse transcribed to cDNA using a GoScript^TM^ Reverse Transcription kit (Promega, WI, USA). qPCR for Mcl-1 was performed with primers specific for Mcl-1 (forward: 5’-CAGCGACGGCGTAACAAAC-3’, and reverse: 5’-ACAAACCCATCCCAGC- CTCTTT-3’) and β-actin (forward: 5’-CATCGAGCACGGCATVGTCA-3’, and reverse: 5’-TAGCACAGCCTGGATAGCAAC-3’). qPCR was carried out with a GoTaq^®^ qPCR Master Mix kit (Promega, WI, USA).

### Immunoprecipitation

Cells were lysed in RIPA buffer, and then centrifuged to collect the supernatant and the amount of protein was quantified. Equal amounts of protein were precipitated with primary antibody overnight at 4°C, and then protein A/G agarose beads were added at 4°C for 4 h. The immunoprecipitates were washed and collected for Western Blot analysis.

### Transfection of siRNA

Mcl-1 siRNA (5’-CCAAGAAAGCUGCAUCGAAdTdT-3’) and non-silencing siRNA (5’-UUCUCCGAACGUGUCACGUTT-3’) were purchased from Sigma-Aldrich (St Louis, MO, USA). Cells were transfected with siRNA (final concentration of 60 nM) using Lipofectamine^®^ RNAiMAX Reagent (Invitrogen, MA, USA). The efficiency of gene knockdown in cells was verified by real-time RT-PCR and Western blot after 48 h of transfection.

### DNA transfection

PANC-1 cells were seeded in six-well plates. Cells were transfected with 2 μg of a pCMV6-Entry expression plasmid encoding human Mcl-1 under the control of a CMV promoter by Lipofectamine^®^ 3000 Reagent (Invitrogen, MA, USA). The efficiency of gene overexpression in cells was verified by real-time RT-PCR and Western blot after 48 h of transfection.

### Measurement of ATP production

Cells (4×10^3^ cells/well) were seeded in 96-well plates, and then incubated at 37°C with 5% CO_2_. To each well 100 μL CellTiter-Glo^®^ reagent (Promega, WI, USA) was added and incubated with cells at room temperature for 10 min before measurement. The fluorescence signal was recorded by automatic biochemical analyzer.

### Measurement of O_2_ consumption

Cells (9×10^3^ cells/well) were seeded in 96-well seahorse microplate 24 h before measurement in normal DMEM medium. Cells were washed and pre-incubated in Seahorse Assay Medium for 1 h prior to analysis. Oxygen consumption rate (OCAR) was measured on a Seahorse XF96 analyzer(Seahorse Bioscience, MA, USA).

### MTT assay

Cells (3×10^3^cells/well) were seeded onto 96-well plates, MTT was added to a final concentration of 1mM for 4 h, and formazan crystals were solubilized by addition of 10%SDS in 10mM HCL. The optical density of each well was measured with a visible microplate reader (Molecular Devices, Sunnyvale, CA, USA) at 570 nm.

### Statistical methods

All data were normalized to control values of each assay and were presented as the mean ± standard deviation (SD). Data were analyzed by a one-way ANOVA or t-tests by using GraphPad Prism version 6.0. Significance was chosen as *P*<0.05.

## Results

### Chidamide promotes Mcl-1 degradation via the ubiquitin-proteasome pathway

**Fig 1:**
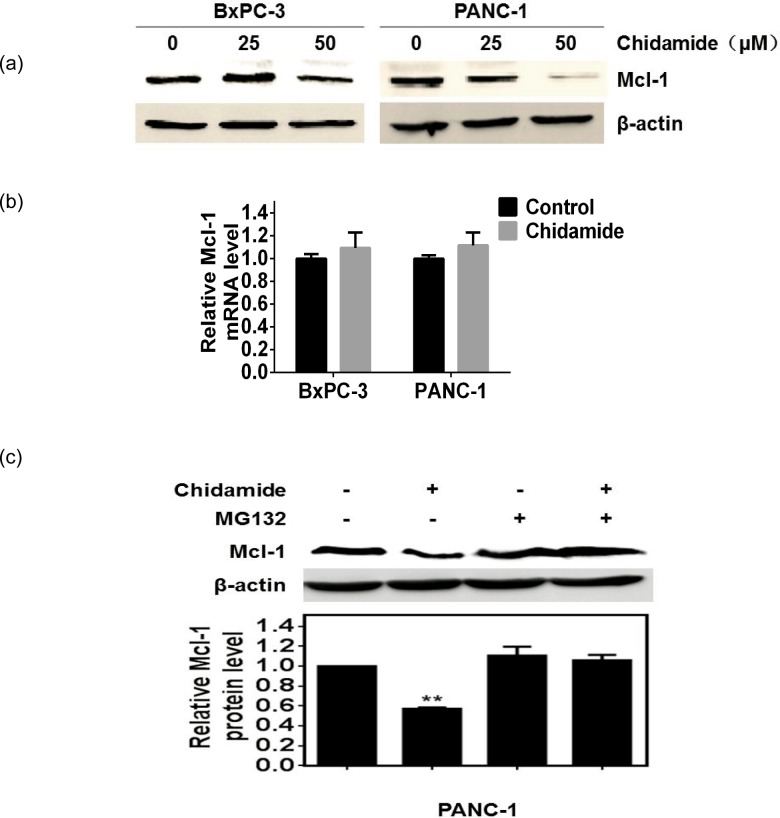
Chidamide promotes Mcl-1 degradation via the ubiquitin-proteasome pathway. (A) Level of Mcl-1 protein in pancreatic cancer cells treated with chidamide for 24 h, determined by Western blot analysis. β-actin served as a loading control. (B) Transcript level of Mcl-1 in pancreatic cancer cells treated with chidamide for 24 h, determined by quantitative real-time PCR. T-tests, BxPC-3: *P* = 0.3097; PANC-1: *P* = 0.1599. (C) MG132 attenuates the degradation of Mcl-1 by chidamide treatment. Western blot was performed to analyze the status of Mcl-1 and β-actin. One-way ANOVA, ** *P*<0.01.

We firstly investigated the effect of chidamide on Mcl-1 expression in BxPC-3 and PANC-1 pancreatic cancer cell lines. BxPC-3 and PANC-1 cells were treated with different concentrations of chidamide (0–50 μM) for 24 h. We found that chidamide treatment decreased the protein level of Mcl-1 in BxPC-3 and PANC-1 cell lines, as measured by Western blot assay (Fig 1A). In contrast, chidamide treatment had no effect on the mRNA levels of Mcl-1 in pancreatic cancer cell lines, as measured by qPCR assay (Fig 1B), suggesting that chidamide decreased Mcl-1 expression via a post-transcriptional process rather than a transcriptional process. Interestingly, a proteasome inhibitor (10 μM MG132) added 6 h before cells were harvested could significantly inhibit the degradation of Mcl-1 by chidamide treatment, as shown in Fig 1C. These results indicate that chidamide may promote the degradation of Mcl-1 via the ubiquitin—proteasome pathway.

### HDACi promotes Mcl-1 protein ubiquitination

**Fig 2:**
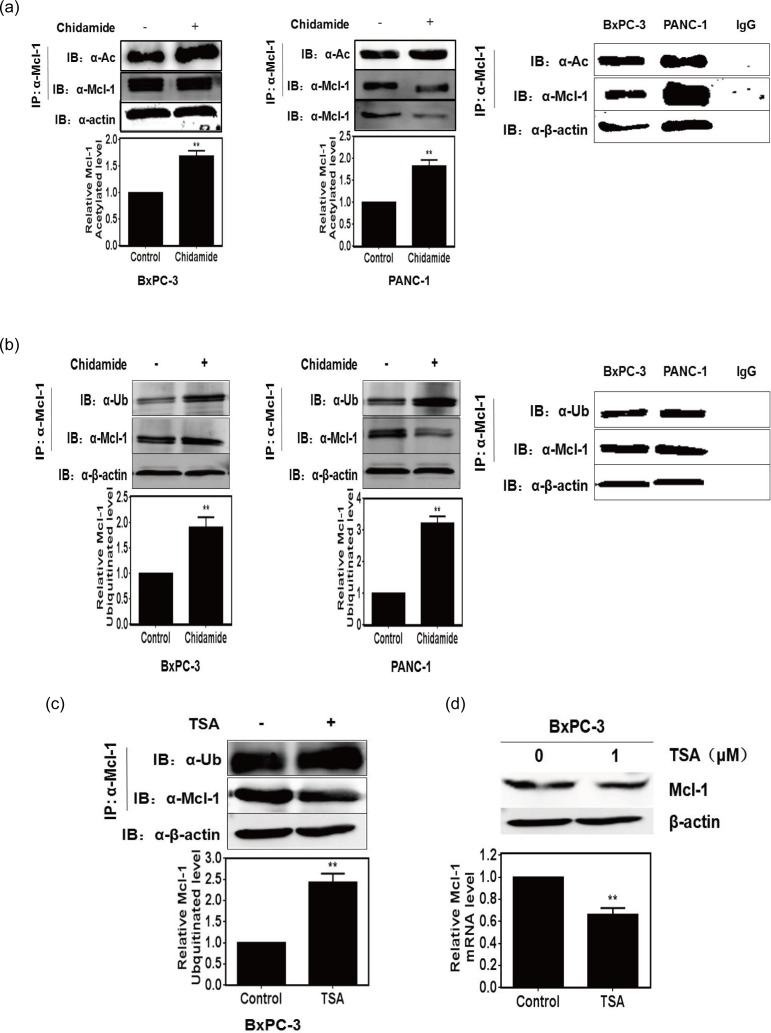
HDACi promotes Mcl-1 protein acetylation and ubiquitination. (A) Acetylated level of Mcl-1 in BxPC-3 and PANC-1 cells treated with chidamide for 24 h, determined by Western blot analysis. (B) Ubiquitinated level of Mcl-1 in BxPC-3 and PANC-1 cells treated with chidamide for 24 h, determined by Western blot analysis. (C) Ubiquitinated level of Mcl-1 in BxPC-3 cells treated with TSA for 24 h, determined by Western blot analysis. (D) Level of Mcl-1 in BxPC-3 cells treated with TSA for 24 h, determined by Western blot analysis. β-actin served as a loading control. T-tests, ** *P*<0.01.

Recent research showed that the HDAC inhibitors (HDACi) promote metabolic protein PEPCK1 degradation through the ubiquitin-proteasome pathway[17]. Moreover Mcl-1 can be ubiquitinated and this ubiquitination affects its stability[18]. At first, we found chidamide, an HDAC inhibitor, significantly increased acetylated level of Mcl-1 in pancreatic cancer cells (Fig 2A). We further assessed whether HDACi regulate Mcl-1 degradation by promoting its ubiquitination. We measured the ubiquitinated level of Mcl-1 in pancreatic cancer cells treated with 50 μM chidamide for 24 h by immunoprecipitation and Western blot analysis. Our results confirmed that chidamide could induce the ubiquitination of Mcl-1 (Fig 2B). We then investigated the effects of another HDACi, Trichostatin A (TSA), on Mcl-1 ubiquitination. After treating the BxPC-3 cells for 24 h with Trichostatin A, immunoprecipitation and Western blot results indicated that TSA also promoted Mcl-1 ubiquitination (Fig 2C) and decreased Mcl-1 protein levels in pancreatic cancer cells (Fig 2D). These results suggest that HDACi could promote Mcl-1 degradation through the ubiquitin-proteasome pathway.

### Chidamide inhibits aerobic metabolism through Mcl-1degradation

**Fig 3:**
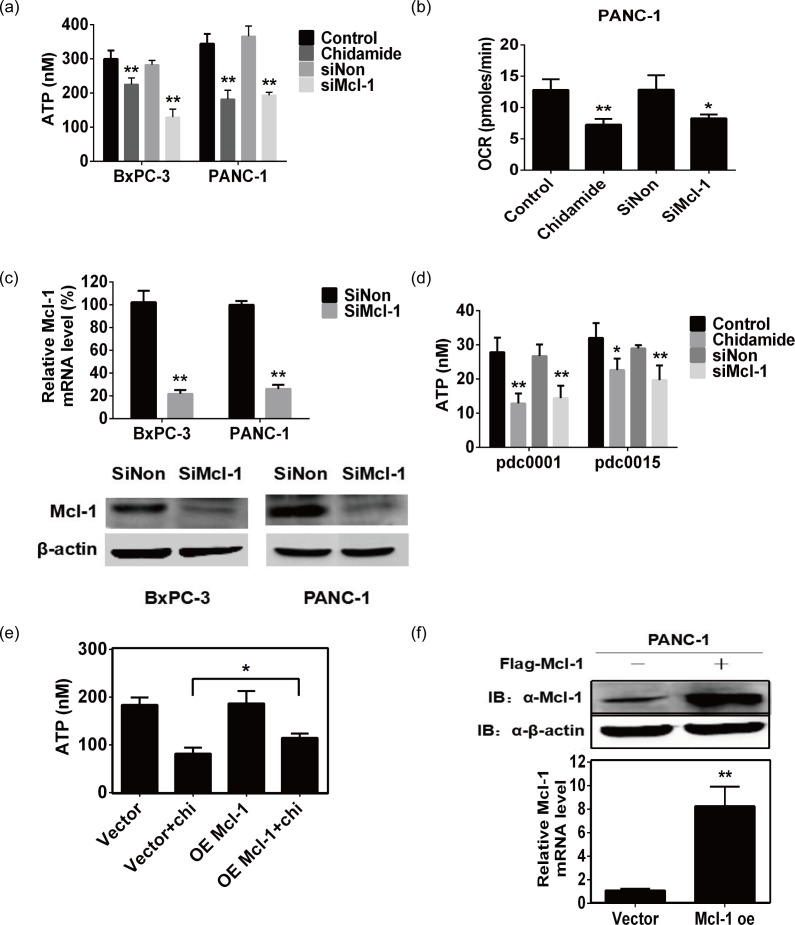
Chidamide inhibits aerobic metabolism through Mcl-1degradation (A) ATP production in pancreatic cancer cells treated with 50 μM chidamide or Mcl-1 siRNA for 24 h, determined by CellTiter-Glo^®^ assay. Data are shown as the means ± SD of three independent experiments. ** *P*<0.01. (B) O_2_ consumption in pancreatic cancer cells treated with 50 μM chidamide or Mcl-1 siRNA for 24 h, determined by Seahorse XF96 analyzer. Data are shown as the means ± SD of three independent experiments. * *P*<0.05, ** *P*<0.01. (C) Interference efficiency of Mcl-1 siRNA was verified by quantitative real-time PCR and Western blot. (D) ATP production in primary pancreatic cancer cells treated with 50 μM chidamide or Mcl-1 siRNA for 24 h, determined by CellTiter-Glo^®^ assay. Data are shown as the means ± SD of three independent experiments. * *P*<0.05, ** *P*<0.01. (E) Overexpression of Mcl-1 attenuates the ATP production inhibition by chidamide in PANC-1 cells, determined by CellTiter-Glo^®^ assay. Data are shown as the means ± SD of three independent experiments. * *P*<0.05. (F) Overexpression of Mcl-1 in PANC-1 cells. PANC-1 cells were transfected with pCMV6-Mcl-1 vector, and subjected to Western blot analysis of Mcl-1 protein level and quantitative real-time PCR analysis of Mcl-1 mRNA level.

The above studies demonstrated the degradation effects on Mcl-1 by chidamide in pancreatic cancer cell lines. In view of the Mcl-1 function in mitochondrial respiration, we next investigated the effects of chidamide on aerobic metabolism. Pancreatic cancer cells were treated with Mcl-1 siRNA or 50 μM chidamide for 24 h, and then the ATP production and O_2_ consumption were measured. CellTiter-Glo^®^ assay results in Fig 3A showed that chidamide decreased ATP production, which was consistent with the effect of Mcl-1 gene silencing. Further, O_2_ consumption monitoring results showed that both chidamide and Mcl-1 knockdown inhibit aerobic metabolism by decreasing O_2_ consumption (Fig 3B). The interference efficiency of Mcl-1 siRNA was verified by qPCR and Western blot analysis (Fig 3C). We obtained similar results for ATP production in primary pancreatic cancer cells (Fig 3D). Next, we investigated the effect of Mcl-1 overexpression in PANC-1 cells on inhibition of the respiration rate induced by 50 μM chidamide. The CellTiter-Glo^®^ assay results in Fig 3E showed that the inhibition of ATP production induced by 50 μM chidamide was attenuated by Mcl-1 overexpression, and efficiency of Mcl-1 transfection was verified by qPCR and Western blot assay (Fig 3F). These results above confirmed the important role of Mcl-1 in maintaining oxidative metabolism, and suggested that chidamide may inhibit aerobic metabolism via suppressing Mcl-1 expression.

### Aerobic metabolic inhibition affects cancer cell proliferation

**Fig 4:**
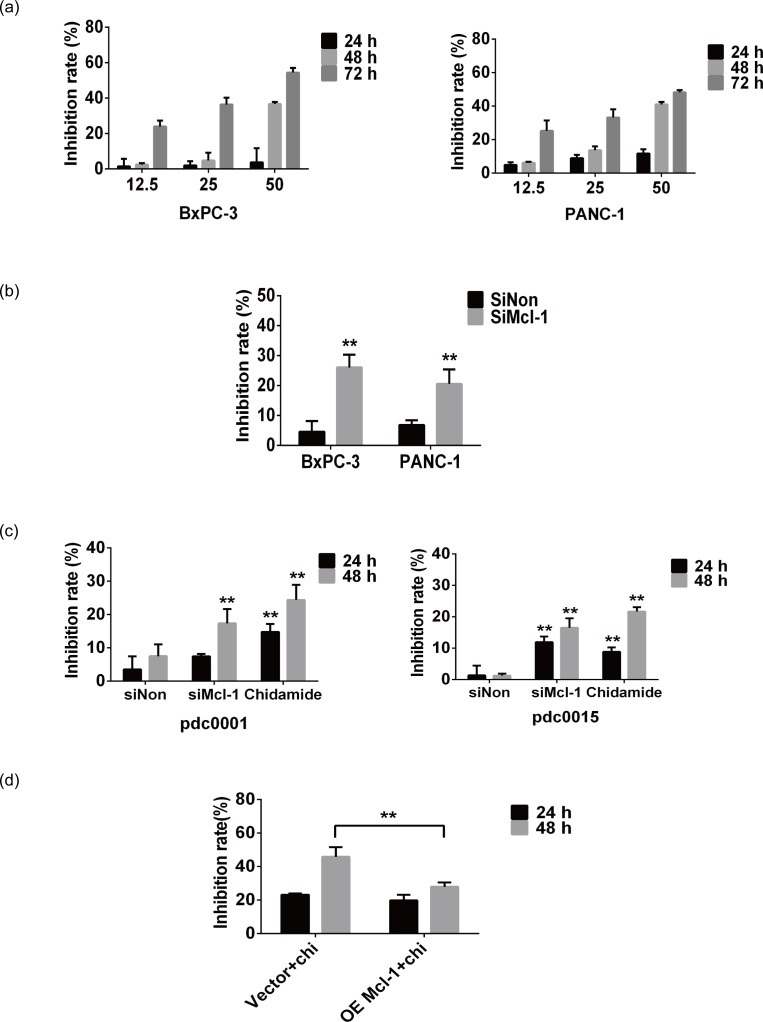
Aerobic metabolic inhibition affects cancer cell proliferation. (A) Pancreatic cancer cells were treated with various concentrations of chidamide for indicated time, proliferation inhibition was determined by MTT assay. (B) Pancreatic cancer cells were treated with Mcl-1 siRNA for 48 h, proliferation inhibition of pancreatic cancer cells was determined by MTT assay. Data are shown as the means ± SD of three independent experiments. ** *P*<0.01. (C) Primary pancreatic cancer cells were treated with 50 μM chidamide or Mcl-1 siRNA for indicated time, proliferation inhibition was determined by MTT assay. Data are shown as the means ± SD of three independent experiments. ** *P*<0.01. (D) 50μM chidamide treated PANC-1 cells were transfected with empty vector or Mcl-1 plasmid for indicated time, proliferation inhibition was determined by MTT assay. Data are shown as the means ± SD of three independent experiments. ** *P*<0.01.

Finally, we studied the significance of aerobic respiration inhibition in cancer cell proliferation and investigated the anti-cancer effects of chidamide in pancreatic cancer cell lines. Cells were treated with Mcl-1 siRNA or different concentrations of chidamide (0–50 μM), and cell survival was evaluated by MTT assay. The MTT results showed that chidamide causes cell growth arrest in a dose- and time-dependent manner in BxPC-3 and PANC-1 cancer cell lines (Fig 4A), which is consistent with the inhibitory effects of Mcl-1 knockdown (Fig 4B). These results suggested that chidamide suppresses cell growth by aerobic metabolism inhibition via Mcl-1 degradation. To further test this assumption, we inspected the proliferation inhibition efficiency of chidamide and Mcl-1 siRNA in pancreatic primary cancer cells. The results in Fig 4C supported the assumption as well. Finally, we investigated the effects of Mcl-1 overexpression on proliferation inhibition by 50 μM chidamide. The results in Fig 4D showed that Mcl-1 overexpression attenuated proliferation inhibition of PANC-1cells by chidamide. These results above indicate that aerobic metabolism inhibition suppresses cell proliferation, and that chidamide inhibits cancer cell growth via aerobic metabolism reduction.

## Discussion

In this study, we demonstrate that Mcl-1 plays an important role in both metabolic and survival processes. In addition, the anti-cancer effect of chidamide in pancreatic cancer may relate to regulation of Mcl-1 degradation. Chidamide induces Mcl-1 degradation through the ubiquitin-proteasome pathway, and consequently inhibits the aerobic metabolism and proliferation of pancreatic cancer cells.

The Western blot assay indicates that chidamide potently promotes the degradation of Mcl-1 proteins via the ubiquitin-proteasome pathway. This result is consistent with previous research demonstrating that ubiquitin-proteasome mediated degradation is a common process regulating Mcl-1 expression. In addition, Mcl-1 can be ubiquitinated at K5, K40, K136, K194 and K197[18]. Recent reports find that virtually all metabolic enzymes are acetylated, and HDACi appears to play an important role in metabolic enzyme activity regulation, given that an acetylated protein can promote ubiquitination of itself[17]. Here we speculated that chidamide as a HDACi increases the acetylated level of Mcl-1 proteins, and then acetylated Mcl-1 is ubiquitinated and subsequently degraded by proteasomes. Therefore, function of Mcl-1 is suppressed, and Mcl-1 stability can be controlled by chidamide.

Next we investigated the function of Mcl-1 downregulation by chidamide in pancreatic cancer cells. Experimental results verified that chidamide dose- dependently decreased ATP production by decreasing O_2_ consumption, which was consistent with the effect of Mcl-1 gene silencing. These results indicate that Mcl-1 plays an important role in the process of energy metabolism. In addition, we observed that the ATP production inhibition induced by chidamide was attenuated by Mcl-1 overexpression. Therefore, chidamide may inhibit aerobic metabolism via suppressing Mcl-1 expression.

In addition to chidamide, other HDACi drugs, such as TSA, have been reported to have anti-cancer activities. Our work agrees with and extends the previous work that chidamide can serve as a potential drug for pancreatic cancer. Previous studies confirmed that chidamide suppresses the PI3K/Akt and MAPK/Ras signaling pathways, arresting pancreatic cancer cells at the G1 phase of the cell cycle and promoting apoptosis[19]. Finally, we inspected the pancreatic cancer cell proliferation influenced by aerobic metabolism. The MTT assay results showed that the proliferation rate was suppressed by chidamide, and the same effect was achieved with Mcl-1 knockdown. We can conclude from these results that aerobic metabolism suppression inhibits cancer cells proliferation rate. In addition, Mcl-1 overexpression attenuated proliferation inhibition by chidamide. Therefore, we speculate that chidamide inhibits cancer cell growth via aerobic metabolism reduction.

Our data indicate another underlying anti-cancer molecular mechanism of chidamide in pancreatic cancer. Mcl-1 is highly expressed in pancreatic cancer cells[7], and its function in mitochondrial aerobic metabolism has been demonstrated. Chidamide promotes Mcl-1 degradation via the ubiquitin-proteasome pathway, and inhibits both ATP production in mitochondria and anti-apoptotic function of Mcl-1. Consequently, chidamide suppresses the energy supply that pancreatic cancer cell proliferation largely demands, and synergistically promotes cancer cells apoptosis.

In conclusion, we verified that Mcl-1 is a chidamide target protein. Chidamide exerts anti-tumor effects by inducing Mcl-1 degradation via the ubiquitin- proteasome pathway, promoting apoptosis and inhibiting aerobic respiration of pancreatic cancer cells. This is the first report of the effect of energy metabolism modulation on proliferation by chidamide through Mcl-1 degradation. We have elucidated an underlying anti-cancer pathway by chidamide. Furthermore, our work suggests that the novel HDACi chidamide has antitumor potential against pancreatic cancer.
